# Peripheral Brain Derived Neurotrophic Factor Precursor Regulates Pain as an Inflammatory Mediator

**DOI:** 10.1038/srep27171

**Published:** 2016-06-02

**Authors:** Cong Luo, Xiao-Lin Zhong, Fiona H. Zhou, Jia-yi Li, Pei Zhou, Jun-Mei Xu, Bo Song, Chang-Qi Li, Xin-Fu Zhou, Ru-Ping Dai

**Affiliations:** 1 Department of Anesthesiology, The Second Xiang-Ya Hospital of Central South University, Changsha 410000, Hunan, China; 2 Department of Anatomy and Neurobiology, School of Basic Medical of Science, Central South University, Changsha 410000, Hunan, China; 3 School of Pharmacy and Medical Sciences, Division of Health Sciences, University of South Australia, Adelaide, SA 5000, Australia; 4 Shanghai Yile Biotechnology Co., Ltd. 466 Yindu Road, Shanghai 200231, P.R.China

## Abstract

The precursor of brain derived neurotrophic factor (proBDNF), the unprocessed BDNF gene product, binds to its receptors and exerts the opposing biologic functions of mature BDNF. proBDNF is expressed in the peripheral tissues but the functions of peripheral proBDNF remain elusive. Here we showed that proBDNF and its predominant receptor, p75 pan-neurotrophin receptor were upregulated in the nerve fibers and inflammatory cells in the local tissue in inflammatory pain. Neutralization of proBDNF by polyclonal antibody attenuated pain in different models of inflammatory pain. Unilateral intra-plantar supplementation of proBDNF by injecting exogenous proBDNF or ectopic overexpression resulted in pain hypersensitivity and induced spinal phosphorylated extracellular signal-regulated kinase activation. Exogenous proBDNF injection induced the infiltration of inflammatory cells and the activation of proinflammatory cytokines, suggesting that inflammatory reaction contributed to the pro-algesic effect of proBDNF. Finally, we generated monoclonal anti-proBDNF antibody that could biologically block proBDNF. Administration of monoclonal Ab-proBDNF attenuated various types of inflammatory pain and surgical pain. Thus, peripheral proBDNF is a potential pain mediator and anti-proBDNF pretreatment may alleviate the development of inflammatory pain.

Brain derived neurotrophic factor (BDNF) is a neurotrophin playing multiple biologic roles including neuronal survival, shaping neurons and synaptic plasticity^1^. Like other neurotrophins, BDNF is initially synthesized as a precursor, which is then cleaved by proteases to mature BDNF (mBDNF)^2^,^3^,^4^. The prodomain of BDNF is essential for the correcting folding and secretory pathway of mBDNF. Not only as an intermediate during the synthesis of mBDNF, proBDNF also binds to its receptors, p75 pan-neurotrophin receptor (p75NTR) and sortilin, and mainly exerts opposing biologic effects of mBDNF in the central nervous system. For example, in contrast to mBDNF, proBDNF can play an active role in neuronal apoptosis, axon pruning and negatively regulates hippocampal dendritic complexity and spine density^5^,^6^.

In addition to the abundant expression in the central nervous system, proBDNF is also expressed in the peripheral nervous system and other tissues such as skin, olfactory epithelium and intestine^7^. In the skin, proBDNF immunoreactivity was observed in the keratinocytes and nerve fibers; and in the intestine, proBDNF was located in the myenteric plexus layer and in the mucosal and submucosal layers^7^. Despite the extensive studies about the role of proBDNF in the central nervous system, the biologic roles of proBDNF in the peripheral tissues still remain elusive. Here, we reported that proBDNF in the peripheral tissues is an inflammatory mediator regulating the inflammatory pain. Anti-proBDNF antibody could attenuate various types of inflammatory pain and may be a potential candidate to ameliorate the inflammatory pain.

## Results and Discussion

**Figure 1:**
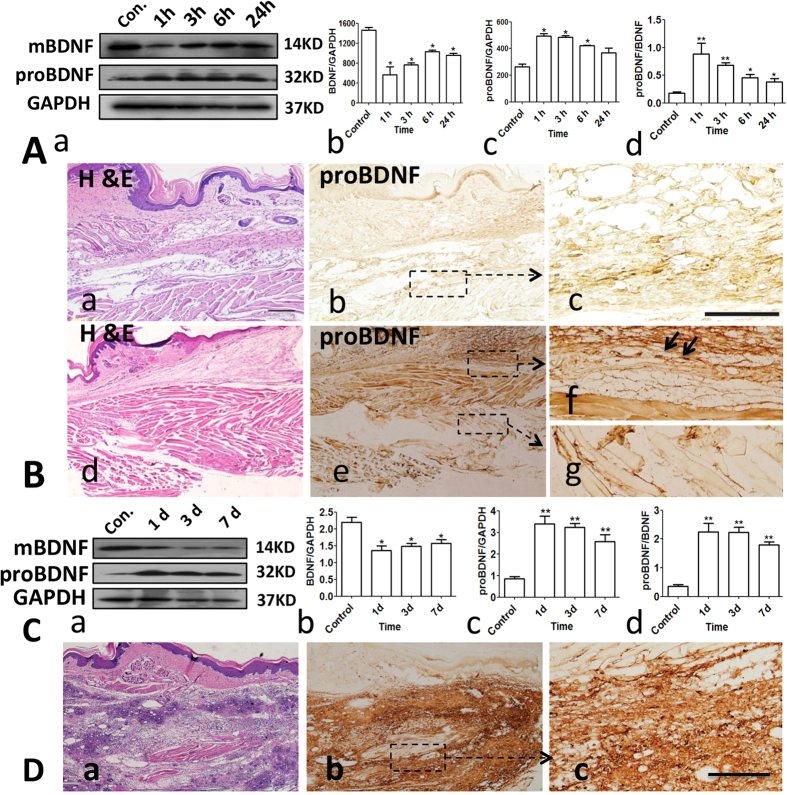
Upregulation of proBDNF in the local tissue in acute and persistent inflammatory pain in mice. (**A**) Representative Western blot (a) and their semi-quantitative analyses of mature BDNF (b), proBDNF (c) and their ratio (d) in the local tissue after 10 μL 5% formalin intra-plantar injection into Kunming mice (*p < 0.05, **p < 0.01 versus control, one-way ANOVA followed by Dunnett’s Multiple Comparison *post hoc* test, n = 3 per group). (**B**) H&E staining (a and d) and immunohistochemsitry (b,c and e–g) of proBDNF in the foot skin at 3 h post-formalin injection. proBDNF is expressed in the epidermis, basal layer and subcutaneous layers in the foot skin (b,c); Higher magnification (box in b) showing proBDNF is also mildly expressed in the nerve fibers in the control plantar (c); Responding to peripheral inflammation by 5% formalin intra-plantar injection, intensive proBDNF immunoreactivity is observed and mainly localized in the inflammatory cells (f, black arrows) and nerve fiber-like structures (g). Scale bars: 50 μm, 3 replicates, n = 3 per group. (**C**) a, Representative Western blot of proBDNF and mBDNF; b–d, Semi-quantitative analyses of mBDNF, proBDNF and their ratio in the inflamed tissue after Complete Freund Adjuvant (CFA, 10 μL) intra-plantar injection into Kunming mice (*p < 0.05, **p < 0.01versus control, one-way ANOVA followed by Dunnett’s Multiple Comparison *post hoc* test, n = 3 per group). (**D**) Histological staining (a) and proBDNF immunohistochemistry (b,c) in the plantar at 1 day post-CFA injection; c, higher magnification of box in b showing that proBDNF is highly expressed in the inflammatory cells. Scale bar, 100 μm, 3 replicates, n = 3 per group. Data bars represent mean ± s.e.m.

Consistent with our previous study^7^, both mBDNF and proBDNF expression was detected in the footpad of Kunming mouse (Fig. 1A). The proBDNF immunoreactivity was distributed in the nerve fibers in the subcutaneous layer (Fig. 1B a–c). Following unilateral formalin intra-plantar injection, proBDNF expression was upregulated, but mBDNF expression was downregulated in injected area (Fig. 1A). The increased proBDNF could be detected in the nerve fibers (Fig. 1B d–g) since abundant proBDNF positive staining was co-localized with the markers of nerve fibers, neurofilament-200 and protein gene product (PGP) 9.5 (See Supplementary Fig. S1). Interestingly, the upregulated proBDNF was also highly distributed in the inflammatory cells responding to peripheral inflammation. Indeed, double labeling immunofluorescence showed that the increased proBDNF was also co-localized with interleukin (IL)-1β and IL-6 positive staining (See Supplementary Fig. S2). Similarly, in the complete Freund’s adjuvant injection-evoked peripheral inflammation, proBDNF was greatly elevated, but mBDNF was greatly downregulated in the injected area (Fig. 1C a–d). The increased proBDNF was also abundantly expressed in the inflammatory cells (Fig. 1D a–c). These findings suggested that peripheral inflammation inhibited proBDNF converting to mBDNF in the local tissue and inflammatory cells were likely to be the important source of the upregulated proBDNF.

**Figure 2:**
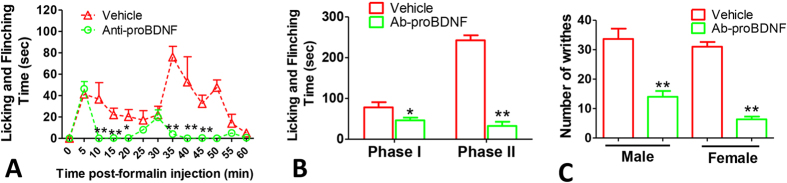
Polyclonal Ab-proBDNF pretreatment attenuates inflammatory pain in mice. (**A,B**) proBDNF polyclonal antibody (5 ml/Kg) *i.p* pretreatment attenuated both phases of nociceptive responses induced by 5% formalin intra-plantar injection in Kunming mice. (**A**) Time course of biphasic nociceptive response (*P < 0.05, **p < 0.01 versus vehicle, two-way ANOVA with Bonferroni’s Multiple Comparison *post hoc* test, n = 8 per group); (**B**) 1^st^- and 2^nd^- phase (*P < 0.05, **p < 0.01 versus vehicle, student’s t test, n = 8 per group). (**C**) Attenuation of abdominal writhing both in male and female Kunming mice by poly-Ab-proBDNF pretreatment (**p < 0.01 versus vehicle, student’s t test, n = 12 per group).

It was well known that mBDNF in the spinal cord is involved in inflammatory and neuropathic pain, and neutralizing the increased spinal mBDNF could attenuate pain processing^8^. The upregulated local proBDNF after peripheral inflammation suggested that peripheral proBDNF may also be involved in the inflammatory pain. In attempt to test this hypothesis, we firstly examined whether neutralizing the increased proBDNF could attenuate formalin injection-evoked inflammatory pain. Formalin intra-plantar injection usually causes a two-phase nociceptive response, the first phase begins immediately after the injection of formalin and lasts for 5 min and the second phase begins from 10 min and lasts for 40–60 min post-injection^9^. Polyclonal anti-human proBDNF antibody (poly-Ab-proBDNF) used in this experiment was proved to specifically and fully block the function of proBDNF (5 ml/Kg)^10^,^11^. Poly-Ab-proBDNF was administered at 30 min before formalin intra-plantar injection. Pretreatment by poly-Ab-proBDNF greatly attenuated both phases of formalin-evoked biphasic nocifensive response (Fig. 2A,B). Furthermore, we studied whether poly-Ab-proBDNF pretreatment also ameliorated inflammatory visceral pain. Visceral pain was established by acetic acid intraperitoneal (*i.p*) injection and evaluated by the number of abdominal writhes^12^. As shown in Fig. 2C, poly-Ab-proBDNF dramatically inhibited visceral inflammatory pain in both sexes of mice as indicated by the dramatic reduction of the number of abdominal writhes. These findings suggest that neutralizing the endogenous increased proBDNF attenuate the inflammatory pain.

**Figure 3:**
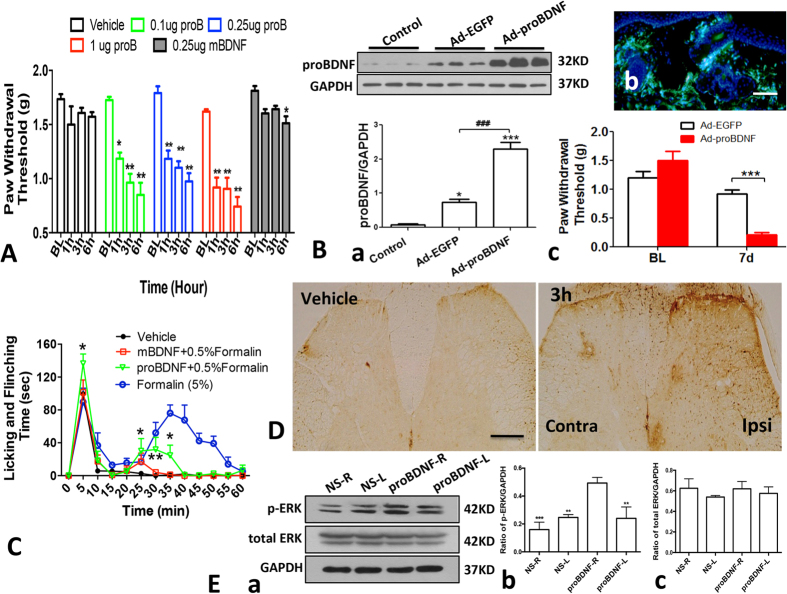
Exogenous proBDNF induces pain hypersensitivity and spinal cord activation in mice. (**A**) Dosage effect of exogenous proBDNF protein on PWT by injection of proBDNF protein into the plantar (*P < 0.05, **p < 0.01 versus baseline, one-way ANOVA followed by Dunnett’s Multiple Comparison *post hoc* test, n = 10–12 per group). (**B**) Ectopic overexpression of proBDNF by intra-plantar injection of *Ad-proBDNF* or *Ad-EGFP* reduces PWT dramatically in Kunming mice. (a) Representative proBDNF Western blot (upper panel) and its semi-quantitative analysis (lower panel, *P < 0.05, ***p < 0.001 versus control, ^###^p < 0.001 versus indicated groups, one-way ANOVA followed by Tukey’s Multiple Comparison *post hoc* test, n = 4 per group); (b) Representative fluorescent images after delivery of *Ad-EGFP*. Scale bar, 50 μm, 3 replicates, n = 3 per group; (c) PWT at 7 days post-injection of *Ad-proBDNF* or *Ad-EGFP* control (***p < 0.001 versus *Ad-EGFP*, student’s t test, n = 7 per group). (**C**) Co-injection of proBDNF (0.1 μg), but not mBDNF (0.1 μg) restored the biphasic nociceptive response after low-concentration of formalin (0.5%) intra-plantar injection (*p < 0.05versus vehicle, two-way ANOVA followed by Bonferroni’s Multiple Comparison post hoc test, n = 10–12 per group). (**D**) Exogenous proBDNF (1 μg) intra-plantar injection induces ERK activation in the ipsilateral spinal cord dorsal horn at 3 h post-injection, Scale bar, 50 μm, n = 3 per group, 3 replicates. (**E**) Spinal p-ERK expression at 3 h after proBDNF (1 μg) intra-plantar injection. (a) Representative Western blot and (b,c) their semi-quantitative analyses of p-ERK, (*p < 0.05, **p < 0.01 versus proBDNF-R, one-way ANOVA followed by Dunnett’s Multiple Comparison *post hoc* test, n = 3 per group). Data bars represent mean ± s.e.m.

Next, we investigated whether the upregulation of proBDNF contributed to pain hypersensitivity. Firstly, we injected recombinant human proBDNF protein into the mice plantar and examined paw withdrawal threshold (PWT) after injection. Recombinant proBDNF intra-plantar injection greatly reduced PWT detected at the dose of 0.1 μg and becoming more robust at dose of 0.25 μg and 1 μg (Fig. 3A). However, mBDNF (0.25 μg) had only marginal effect on PWT (Fig. 3A). These results suggest that peripheral proBDNF, but not mBDNF, induces the mechanical pain hypersensitivity. To further verify whether upregulated proBDNF induces mechanical hypersensitivity, we examined the effect of over-expressing peripheral proBDNF on PWT. Adenovirus vector encoding proBDNF gene and enhanced green fluorescent protein gene *(Ad-proBDNF*), and the control adenovirus vector which only carrying EGFP gene (*Ad-EGFP*) were delivered into the footpad of mice. At 7 days post-injection, high expression of proBDNF was detected by EGFP fluorescence and immunoblot (Fig. 3B a,b). Delivery of *Ad-EGFP* decreased PWT slightly, properly due to the non-specific inflammation. In contrast, overexpression of proBDNF by intra-plantar injection of *Ad-proBDNF* dramatically decreased PWT (Fig. 3B c), supporting the assumption that proBDNF renderers the pain hypersensitivity. Lastly, we examined whether proBDNF also contributed to the inflammatory spontaneous pain. Lower concentration of formalin (0.5%) only rendered the first phase of nociceptive response. We injected recombinant human proBDNF (0.1 μg) or mBDNF (0.1 μg) with 0.5% formalin together to examine the nociceptive response. Co-injection of 0.5% formalin with proBDNF could restore the second phase of nociceptive response. However, co-injection of mBDNF with 0.5% formalin had no such effect (Fig. 3C). These findings suggest that exogenous local proBDNF, but not mBDNF, exacerbates spontaneous pain.

If peripheral proBDNF contributed to pain processing, it should lead to spinal activation. Phosphorylated extracellular signal regulated kinase (p-ERK) in the spinal cord is an indicator of spinal nociceptive processing in pain^13^. Therefore, we examined the effect of exogenous proBDNF injection on p-ERK expression in the spinal cord. As shown in Fig. 3D,E, unilateral intra-plantar injection of proBDNF (1 μg) greatly increased the p-ERK expression in the ipsilateral dorsal horn. These findings further strengthen the hypothesis that peripheral proBDNF is a pain mediator.

**Figure 4:**
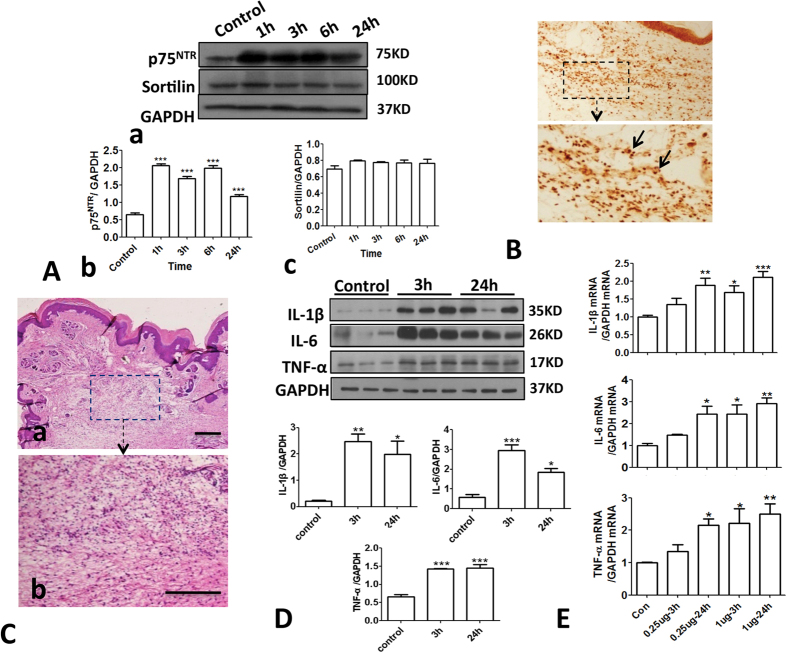
Upregulation of p75NTR and effect of local proBDNF injection on inflammatory reaction in mice. (**A**) Representative Western blot of p75NTR and sortilin (**a**) and the semi-quantitative analysis of their expression (b and c) after formalin intra-plantar injection (***p < 0.001 versus control, one-way ANOVA followed by Dunnett’s Multiple Comparison *post hoc* test, n = 3 per group). (**B**) p75NTR immunohistochemistry in the local tissue at 3 h after 5% formalin intra-plantar injection. Image in lower panel is the higher magnification of box in the upper panel. Scale bar, 50 μm, 3 replicates, n = 3 per group. (**C**) H&E staining of local tissue 3 h after exogenous proBDNF (1 μg) injection, Scale bars, 100 μm, 3 replicates, n = 3 per group. (**D**) Representative Western blot of local cytokines IL-1β, IL-6 and TNF-α expression and their semi-quantitative analysis at 3 h and 24 h after 1 μg exogenous proBDNF intra-plantar injection (*p < 0.05, **p < 0.01, ***p < 0.001 versus control, one-way ANOVA followed by Dunnett’s Multiple Comparison *post hoc* test, n = 4 per group). (**E**) Time course of IL-1β, IL-6 and TNF-α gene expression 3h after 0.25 μg and 1 μg proBDNF injection (*p < 0.05, **p < 0.01, ***p < 0.001 versus control, one-way ANOVA followed by Dunnett’s Multiple Comparison *post hoc* test, n = 3 per group). Data bars represent mean ± s.e.m.

In the peripheral inflammation, the increased proBDNF was highly expressed in inflammatory cells abundantly co-localized with IL-1β and IL-6 positive cells (See Supplementary Fig. S2). Given that proBDNF exerted its biologic functions through acting on its receptors, p75NTR and sortilin, we examined whether inflammation also activated p75NTR and sortilin in the local tissue. In response to inflammation, p75NTR, but not sortilin, was greatly increased and widely distributed in the inflammatory cells (Fig. 4A,B, also see Supplementary Fig. S3). Previous studies have shown that p75NTR death domain could interact with receptor-interacting protein-2 and resulted in NF-κB activation, a well-known transcription factor regulating proinflammatory cytokine gene expression^14^,^15^. Thus, the activated proBDNF-p75NTR signaling may exert its pro-algesic effect through the inflammatory reaction. To test this hypothesis, we injected recombinant human proBDNF protein (1 μg) into the mice plantar and examined its effect on inflammatory response. Indeed, after exogenous proBDNF intra-plantar injection, abundant inflammatory cells were recruited into the injected zone (Fig. 4C). We further examined the local proinflammatory cytokines including IL-1β, IL-6 and tumor necrosis factor (TNF)-α levels after proBDNF intra-plantar injection. Recombinant proBDNF injection elevated IL-1β, IL-6 and TNF-α expression (Fig. 4D and Supplementary Fig. S4). These findings suggested that proBDNF injection resulted in the proinflammatory response. Consistently, the local proinflammatory cytokines gene expression was also activated in response to proBDNF intra-plantar injection (Fig. 4E). Taken together, our results strongly indicate that proBDNF exacerbates pain processing through activating the inflammatory reactions.

**Figure 5:**
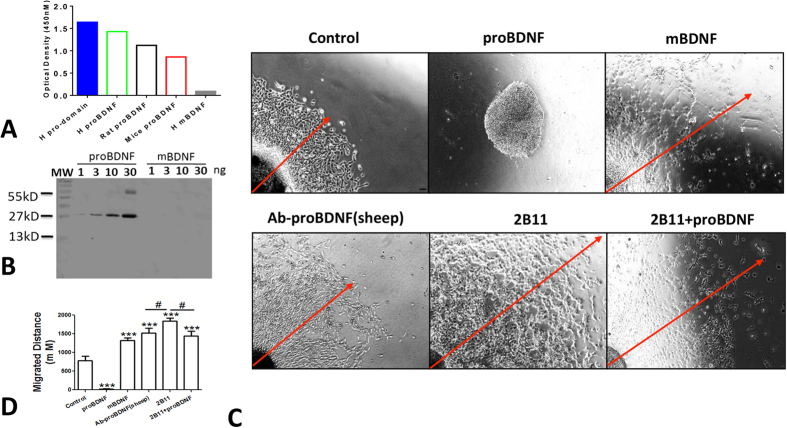
Characterization of proBDNF monoclonal antibody 2B11. (**A**) ELISA assay for the immunoreactivity of 2B11 against human proBDNF prodomain, and human, rat and mice proBDNF proteins, and human mature BDNF (mBDNF). 2B11 has strong immunoreactivity against proBDNF and prodomain, but not mBDNF; (**B**) Representative Western blot of human proBDNF and mBDNF detected by 2B11 (dilution 1:2000), note that 2B11 specifically recognizes proBDNF, but not mBDNF. (**C**) Representative images of neurosphere radiant migration treated by proBDNF, mBDNF, sheep polyclonal anti-proBDNF antibody, mouse monoclonal anti-proBDNF antibody 2B11 and co-treatment. (**D**) Statistical analysis of neurosphere migration radiance assay (***P < 0.001 versus control, ^#^p < 0.05 versus indicated group, one-way ANOVA followed by Tukey’s Multiple Comparison *post hoc* test). Neurospheres treated with proBDNF (100 ng/mL) showed dormancy without any neuronal migration and neurospheres had no morphological changes. Neurospheres treated with 2B11 (100 ng/ml) showed strong migration capability comparing with other groups. Neurospheres treated with 2B11 and proBDNF (100 ng/ml) showed similar ability of migration with sheep anti-proBDNF antibody treatment group.

Monoclonal antibody is widely used to develop the drug to treat diseases including pain^16^,^17^. For example, the monoclonal antibody against nerve growth factor (NGF) has been developed to treat pain with osteoarthritis^18^. The analgesic effect of poly-Ab-proBDNF strongly encouraged us to generate the monoclonal antibody against human proBDNF. The specificity of various monoclonal anti-proBDNF clones were developed and tested by indirect ELISA assay. After screening, the clone 2B11 was selected since it displayed strong immunoreactivity against proBDNF, but not mBDNF. As shown in Fig. 5A, 2B11 had very strong reactivity against the prodomain of human proBDNF, but not mBDNF. Western blot studies also showed that 2B11 only recognized proBDNF, but not mBDNF (Fig. 5B). Thus, the developed monoclonal antibody specifically recognized proBDNF, but not mBDNF. Given that mBDNF could promote the neurosphere outgrowth whereas proBDNF collapses it^11^, the neurosphere radiant migration assay was undertaken to assess the biological effects of the selected monoclonal antibody clone 2B11. Single neurosphere with the similar diameter of 500–600 μm was used for the assay by measuring the migration distance. In the control medium, the migration distance of neurosphere was around 800 μm (Fig. 5C). Treatment by proBDNF (100 ng/ml) totally blocked the neurosphere migration. In contrast, mBDNF treatment promoted the neurosphere migration. Treatment by poly-Ab-proBDNF or 2B11 also greatly promoted the neurosphere outgrowth. More importantly, 2B11 treatment could fully block the neurosphere collapse induced by proBDNF (Fig. 5C,D). Thus, the 2B11 clone could functionally block the effect of proBDNF. Taken together, the developed monoclonal antibody 2B11 specifically recognize the proBDNF domain in various species and block the biologic effect of proBDNF.

**Figure 6:**
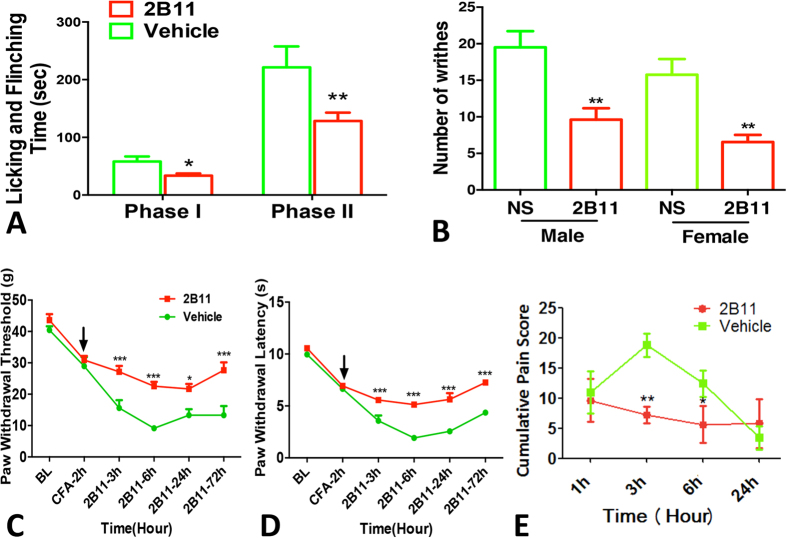
Effect of 2B11 on different types of inflammatory pain. (**A**) Preventive effect of 2B11 on 5% formalin hindpaw injection-induced nociceptive response of both the 1^st^- and 2^nd^- phase in male Kunming mice (*p < 0.05, **p < 0.01versus vehicle, student’s t test, n = 10 in vehicle group, n = 12 in 2B11 group). (**B**) 2B11 reduced the number of writhes in visceral pain in male and female Kunming mice (**p < 0.01versus vehicle, student’s t test, n = 18 in NS group, n = 20 in 2B11 group). (**C,D**) Therapeutic effect of 2B11 on persistent inflammatory pain. (**C**) Paw withdrawal threshold (PWT) and (**D**) paw withdrawal latency (PWL). 2B11 was administered by *i.p.* injection at 2 h post CFA-injection in SD rats (**p < 0.01, ***p < 0.001versus saline control, two-way ANOVA followed by Bonferroni’s Multiple Comparison *post hoc* test, n = 8 per group). (**E**) 2B11 *i.p.* pretreatment reduced incision-induced surgical pain in male SD rats (*p < 0.05, **p < 0.01versus vehicle, two-way ANOVA followed by Bonferroni’s Multiple Comparison *post hoc* test, n = 8 per group). Data bars represent mean ± s.e.m.

Next, we examined whether the clone 2B11 could inhibit the experimental inflammatory pain model. The clone 2B11 (5 mg/kg) was administered by *i.p* injection at 30 min before formalin intra-plantar injection. 2B11 could greatly inhibit the biphasic nociceptive response to formalin injection (Fig. 6A). In addition, 2B11 pretreatment could greatly inhibit inflammatory visceral pain as indicated by reducing the number of writhes responding to acetic acid *i.p* injection (Fig. 6B). These findings suggested that administration of mono-Ab-proBDNF could prevent the incidence of acute inflammatory pain. To further examine the therapeutic effect of mono-Ab-proBDNF on inflammatory pain, CFA intra-plantar injection was conducted in the Sprague-Dawley (SD) rats to induce persistent pain. At 2 hour post-injection, mechanical allodynia and thermal hyperalgesia were developed as indicated by the decrease of paw withdrawal threshold (PWT) and paw withdrawal latency (PWL), *i.p* administration of 2B11 (5 mg/kg) greatly attenuated the reduced PWT and PWL at 3 hour to 72 hour post-injection (Fig. 6C,D), suggesting the therapeutic effect of mono-Ab-proBDNF. Finally, we investigated whether 2B11 also ameliorated other types of pain. Surgical pain was established by hindpaw incision in the SD rats as described previously^19^ and clone 2B11 (5 mg/kg) was delivered by *i.p* injection at 30 min before surgery. 2B11 pretreatment could greatly inhibit the pain score after hindpaw incision indicating that 2B11 also inhibits surgical pain (Fig. 6E). Thus, monoclonal anti-proBDNF antibody may be a novel potential drug to prevent and treat the progress of pain.

In the present study, we have showed the novel function of proBDNF, *i.e.*, peripheral proBDNF regulated pain. Accumulating evidence has shown that mBDNF regulated pain through central sensitization. In inflammatory pain, spinal mBDNF is upregulated and mediates inflammatory pain processing^20^,^21^. Furthermore, spinal mBDNF secreted from microglia also regulate mechanical allodynia in the neuropathic pain^22^,^23^. In a sharp contrast, proBDNF may mediate pain processing through peripheral sensitization. In particular, the abundant expression of proBDNF and p75NTR in nerve fibers and inflammatory cells suggests that proBDNF not only directly acts on primary sensory neurons but also indirectly activates inflammation which contributes to the pro-algesic effect of proBDNF. However, the analgesic effects of Ab-proBDNF are mainly limited in the acute inflammatory pain in the present study. Further studies are needed to investigate the effect and its underlying mechanism of Ab-proBDNF on the chronic phase of pain, which may be more relevant to the clinical practice.

Neurotrophins have now been suggested as the potential target to treat pain^8^,^24^. Monoclonal antibody against NGF (such as Tanezumab) has been proven to be effective for osteoarthritis pain and still actively undertaken the clinical trial^25^. However, the potential bone degeneration of Tanezumab could be compromised for its clinical application^26^. The effect of mBDNF on pain mainly through spinal cord mechanisms may require the intrathecal injection and thus limits its potential clinical application. The developed 2B11 ameliorated different types of inflammatory pain and surgical pain through systemic administration suggest that it may be a potential therapeutic drug to treat pain. More importantly, proBDNF is a normal intracellular intermediate and therefore Ab-proBDNF injection may not render many severe side effects in physiological conditions. Thus, the developed monoclonal antibody may be a potential intervention to prevent the development of pain processing.

## Methods

### Animals

There are total 197 male and 50 female Kunming-mice (age 6–8 weeks), 32 male Sprague Dawley (SD) rats (Weight 200–250 g) obtained from Central South University Animal Service (Changsha, China). Twenty male C57 B/L mice (embryonic day 14) were bred in Reid Animal Facility, School of Pharmacy and Medical Sciences University of South Australia. All animals are housed in constant temperature (25 °C) and humidity (50%) conditions at 12 h:12 h light/dark cycle and with free accessing to food and water. The experiments were approved by the Animal Care and Use Committee of Central South University and according to the National Institutes of Health Guide for the Care and Use of Laboratory Animals.

### Generation of polyclonal and monoclonal antibodies against prodomain of proBDNF

The production of polyclonal anti-human proBDNF antibody has been described in our previous studies^10^,^11^. Briefly, peptide sequence 69–82 of the human BDNF gene conjugated with keyhole limpet hemocyanin (KLH) or the recombinant prodomain fragment made from *E. coli* were used for immunization in rabbits and sheep (n = 2). The antibodies were affinity purified against the immunizing peptide which was immobilized on Sepharose 4B gel.

For the generation of monoclonal anti-human proBDNF, the entire human amino acid sequence of proBDNF was subcloned in pET22b (Novagen, EMD Biosciences) using EcoRI/XhoI sites to construct pET22b-proBDNF. The proBDNF protein was expressed in BL21 (DE3) bacterial cells and purified as described previously^2^. Purified proBDNF protein was used to immunize mice as well as to screen hybridoma clones. Clone 2B11 was selected from the initial screening and further characterized with ELISA and Western Blot.

### Recombinant mBDNF and proBDNF

Production of recombinant mBDNF and proBDNF used for the bioassay of antibody was made from SF9 insect cells by Virovek (http://www.virovek.com/) on the basis of contract research and well characterized in our previous studies^7^,^10^,^11^,^27^.

The recombinant rat proBDNF and human proBDNF prodomain for ELISA assay were generated by cloning the entire amino acid sequence of rat proBDNF or the sequence of human proBDNF prodomain into pET22b (Novagen, EMD Biosciences) using EcoRI/XhoI sites. The proBDNF or prodomain protein were expressed and purified in BL21 (DE3) bacterial cells. Other recombinant human proBDNF, mice proBDNF and human mBDNF for ELISA, Western Blot or behavior studies were purchased from Alomone Labs (Israel).

### Construction, production and purification of adenovirus encoding human proBDNF gene

High titer adenovirus vectors encoding proBDNF gene (*Ad-proBDNF*) were obtained from GeneChem (Shanghai, China). Briefly, the coding sequence of *proBDNF* was amplified by RT-PCR. The PCR fragments and the pDC315-EGFP plasmid were digested with Age I and then construct the pDC315-proBDNF-EGFP plasmid using in-Fusion cloning technology (Clontech® Laboratories, Inc., Mountain View, CA). The plasmid was transformed to competent DH5α *Escherichia coli* for identification. The primers pDC315-F was located in mCMV promoter and the primer EGFP-N-R was located in the N terminal of EGFP in the vector. The primer (pDC315-F and EGFP-N-R) sequences were as follows: forward, 5′-GGTATAAGAGGCGCGACCAG-3′; reverse, 5′-CGTCGCCGTCCAGCTCGACCAG-3′. The expression of proBDNF-EGFP was further confirmed by transfecting the plasmid into 293T cells using Lipofectamine 2000 (Invitrogen, USA). For generation of recombinant adenoviruses (*Ad-proBDNF-EGFP*), the pDC315-proBDNF-EGFP plasmid, which encodes proBDNF-EGFP, was used to co-transfect HEK293 cells with plasmid pBHG lox ΔE1,3 Cre. Transfection solutions were prepared by mixing 5 μg of the pDC315-proBDNF-EGFP plasmid, 5 μg of the pBHG lox ΔE1,3 Cre plasmid and 10 μL Lipofectamine 2000 in 50 μL antibiotics-free DMEM. After 10–15 d, the supernatant was harvested from HEK 293 cells. After 3 rounds of virus amplification, the supernatant was filtered at 0.45 μm, and purified using Adeno-X™ virus purification kit (BD Biosciences, Clontech). After resuspension, serially diluted adenovirus was used to transduce HEK293 cells. After 7 d, labeled HEK293 cells were counted to calculate the viral titer (8.0 × 10^9^ pfu/mL). A fluorescence microscope could observe the effects of transfection *in vivo.*

### Characterization of Ab-proBDNF monoclonal antibody

The purified monoclonal antibody against proBDNF was characterized by indirect ELISA^27^ and Western Blot. For ELISA assay, 96-well polystyrene microtiter plates (Nunc, Roskilde, Denmark, USA) were coated with the generated human proBDNF prodomain, and commercial human, rat and mice proBDNF proteins (Alomone Labs, Israel), and human mBDNF (Alomone Labs, Israel) (all at 1 μg/mL with total volume 50 μL) overnight at 4 °C. The plates were washed three times in PBS–Tween-20 (0.05%, PBS-T), and the unbound sites were blocked with 100 μL of 2.5% skim milk at 37 °C for 1.5 h. The plates were washed as described above. The developed monoclonal anti-proBDNF antibody was added to the wells starting from 1:500 (37 °C for 1.5 h). After washing, HRP-conjugated rabbit anti-mouse immunoglobulin (Ig, 1:1000 v/v, Bio-Rad, Hercules, CA) was added and incubated for 1.5 h at 37 °C. The plates were washed, and the substrate solution containing tetramethylbenzidine (TMB) was added. The reaction was stopped by 20% H_2_SO_4_ after 15 min and the absorbance was measured at 450 nm by an ELISA reader (BioTek, U.S.).

### Formalin-Induced Acute Inflammatory Pain

Fifty male Kunming-mice were used in this experiment. We used the classical formalin test to demonstrate the role of proBDNF on acute inflammatory pain. Each mouse in this experiment was firstly placed in a Plexiglas box which bottom is made up of metal mesh for 30 minutes to eliminate spontaneous explorative behavior. For testing the effect of proBDNF antibody on pain, each mouse was gently restricted and *i.p.* injection of 0.2 ml polyclonal (5 ml/kg)^10^ or monoclonal (5 mg/kg) anti-human proBDNF antibody generated as described above or 0.9% saline, respectively. Thirty minutes later, all of these mice were subjected to the unilateral (right) intra-plantar injection with 10 μl 5% formalin. For investigation of whether exogenous proBDNF would exacerbate pain behavior, each mouse was injected with 10 μl 5% formalin alone or a mixture of 0.5% formalin with 0.1 μg proBDNF or 0.5% formalin with 0.1 μg mBDNF into the plantar aspect of the hindpaw. After that, all these mice were put back to the box immediately and a settled camera directed to the plantar surface of the hindpaws of these mice was used to record their licking activity. An experimenter who is blind to the protocol analyzed the pain scores, which were defined by licking time per 5 min in 1 hour and the total licking time in each of the two phases of 0–10 min and 10–60 min.

### Visceral Pain

Fifty male and fifty female Kunming-mice were used in this experiment to exclude the gender difference of visceral pain. Each mouse in this part was accommodated to an opaque cage for 30 min before the experiment starts. The time and dose of proBDNF antibody used in this test was the same as above. Afterwards, the mice were *i.p.* injected with 0.6% acetic acid to establish visceral pain model as described previously^28^. The visceral pain intensity was judged by number of obvious writhes of abdomen concomitant with the stretch of two hind limbs in a 15 min period immediately after injection by a tester who was blind to the experimental grouping design.

### Persistent inflammatory pain

Sixteen male SD rats were used in this experiment to demonstrate the local effect of proBDNF monoclonal antibody on pain. Each rat was firstly intra-plantar injected with 50 μl Complete Freund’s adjuvant (CFA). At 2 h post-injection, monoclonal Ab-proBDNF 2B11 (5 mg/kg) dissolved in 0.2 mL saline or equal volume of saline alone were administered by *i.p* injection. At the indicated time point, the PWT and PWL were measured by an experimenter who was blind to the group assignment.

### Incisional pain

Sixteen male rats were used to develop acute surgical pain model as introduced before^19^. Specifically, incisional pain was established under 2.5% sevoflurane with 2 L/min oxygen anesthesia. A 1 cm long lengthwise skin incision was made and the plantaris muscle was exposed and a 0.5 cm longitudinal incision was conducted. After that, the skin was carefully sutured and treated with antibiotic ointment. 200 μl monoclonal proBDNF antibody (5 mg/kg) was *i.p.* injected 30 min before incision induction. An experimenter who was blind to the grouping design helped to measure the accumulative pain score (CPS) after incision.

### Paw Withdrawal Threshold

PWT was measured with von frey filament test using the up and down method introduced previously^29^. Specifically, mice were accommodated in independent boxes with metal mesh which placed in a quiet room for 30 minutes to reduce their explorative behaviors. When test begins, a series of filaments represent separate forces which starts from 0.16 g or 2 g were perpendicularly against a mouse’s or a rat’s plantar of hind paw. If the rodent has a positive reaction (apparent withdrawal, licking, jumping), we record it as “X” and a weaker filament was used, if the opposite, we record it as “O” and a stiffer one was then applied until we get a six number sequence of O and X. The finally paw withdrawal threshold can be obtained by a formula offered by Chaplan. The maximum and minimum limitation of this test was set up as 2.0 g and 0.02 g for mice and 15 g and 0.6 g for rats.

### Thermal hyperalgesia

Paw withdrawal latency (PWL) test was used as introduced before^30^. Rats were separately put in boxes with only the bottom is transparent in a quiet room for 30 min to eliminate explorative behavior. Ugo Basile plantar test (Ugo Basile, Italy) was used to measure the PWL. An infrared heat stimulus was applied to the plantar surface of right hand paw of the rat, and the latency of withdrawal of the paw was automatically recorded. The intensity of the stimulus of each rat was determined by setting up the baseline latency about 10 sec. There is a 10 min interval between each stimulus. The tester is unknown about the grouping design.

### Cumulative pain score

Cumulative pain score (CPS) was used to judge the pain intensity after incision^31^. Rats were handled and accommodated similarly to that used for the PWT test. An experimenter who was blind to the grouping design observed and scored the position of the right hindpaw of rats every 5 min for 1 hour after incision, and each observation lasted for 1 min. The pain score was assessed as follows: 0: full weight-bearing of the metal mesh which blanched or distorted the wound. 2: the incision foot is completely lifted off the mesh. 1: the foot touched the mesh with no blanching or distorting. The cumulative score of 0–24 was acquired during the 1 hour period and the higher scores represented more exaggerated pain behavior.

### Immunohistochemistry and Immunofluorescence

Mice in related experiments were anesthetized by overdose chloral hydrate in appropriate time, respectively. Then they were trans-aortic perfused with 0.9% saline followed by 4% paraformaldehyde. The glabrous skin of the hindpaw and the lumbosacral enlargement of the spinal cord were removed respectively and post fixed with the same fixative overnight at 4 °C. Spinal cord samples were then sequentially immersed in 15% and 30% sucrose for cryoprotection, glabrous skin tissues were paraffin-embedded directly. After this, both of them were cut into slices for further immunostaining.

Paraffin-embedded hindpaw skin slices that are 4 μm thick were firstly sequentially immersed in two dimethylbenzene, two 100% ethanol, 95% ethanol, 75% ethanol for 5 min respectively. Then they were completely rinsed with 0.01M PBS. Tissues were next immersed in citric acid (Cwbiotech, Beijing) and handled with water-bath heating for 18 min at 98 °C for antigen retrieval. After this process, they were followed with immunostaining described as below.

Floating spinal cord sections with 30 μm thickness and part of the handled hindpaw skin slices with 25 μm thickness were rendered for immunohistochemistry analysis. Slices were firstly rinsed in 0.01M phosphate buffer saline (PH = 7.4) for 3 times (10 minutes each time). Then they were immersed in 3% H_2_O_2_ in 2% Triton X-100R at room temperature for 30 min to remove endogenous peroxidase. Next, after thoroughly rinsed, slices were blocked with 5% BSA at 37 °C for 1 hour. Primary antibodies included p-ERK, IL-1β, IL-6 (Cell Signaling Technology, Massachusetts, diluted in 1:1000), proBDNF, p75 (Millipore, Boston, diluted in 1:1000) were then incubated at 37 °C for 1 hour and at 4 °C overnight, respectively. The following reagents used the next day were goat anti-rabbit or goat anti-mouse secondary antibody immunoglobulin (Jackson ImmunoResearch Liboratories, Inc, PA, diluted in 1:200) and ABC kit (Vector Laboratories, Burlingame, CA, diluted in 1:200) which were incubated for 1 hour at 37 °C, respectively. Diaminobenzidine tetrahydrochloride (DAB, ZSGB-BIO, Beijing, China) was used for visualization. All sections were dehydrated in series of graded ethanol and xylene.

The other parts of hindpaw skin slices were used for immunofluorescence analysis. All the protocol before secondary antibody incubation is exactly the same with immunohistochemistry staining except for H_2_O_2_ immersing. The donkey anti-rabbit (Alexa Fluor 594) or donkey anti-mouse(Alexa Fluor 488) IgG H&L (Abcam, Cambridge, UK, diluted in 1:1000) secondary antibody were used in this part for 1 hour at 37 °C. After thoroughly rinsed, they were cover slipped with mounting medium (Vector Laboratories, Inc. Burlingame, CA) and visualized by fluorescence optical miroscopy.

### Western Blotting

The detached inflamed tissue in the hindpaw from the mice with different manipulations were homogenized in lysis buffer (CWbiotech, Beijing, China) with 1% protease inhibitor cocktail and 1% EDTA solution. After standing for 20 min, they were centrifuged at 12000 rpm for 20 min at 4 °C. Afterwards, the supernatant was denatured and used for further analysis. A total of 100 μg protein of each samples were loaded and separated by 10% or 15% Bis-Tris SDS-PAGE gel, for further confirm the specificity of 2B11. 1, 3, 10, 30 ng proBDNF or mBDNF (Alomone Labs, Israel) also loaded onto a 10% SDS-PAGE gel. Proteins then transferred to 0.45 μm or 0.22 μm PVDF membrane respectively according to the molecular weight of each target proteins. Each membrane was firstly blocked with 10% defatted milk for 2 hours at room temperature and followed with incubating with separate primary antibodies overnight at 4 °C which introduced above or with 2B11. Membranes were thoroughly rinsed and incubated with HRP-conjugated goat anti-rabbit or goat anti-mouse secondary antibody (Millipore, Boston, diluted in 1:10000) for 2 hours at room temperature. Finally, they were rinsed and exposed to photographic film with chemiluminescent HRP substrate (Millipore, Boston). Western bloting band were analyzed by the mean grey value with NIH Image J 7.0 and standardized to GAPDH control protein.

### RNA extraction, reverse transcription (RT) and real-time PCR

Total RNA was isolated using TRIzolR reagent based on the company protocol (Invitrogen, USA). For reverse transcription, the reaction mixture containing 2 μg of RNA, 2.5 μM of oligo(dT) primer, and five units of Molony Murine Leukemia Virus Reverse Transcriptase (MMLV, Promega, USA) in a total volume of 25 μl, was incubated for one hour at 42 °C, and stopped by heating for 5 minutes at 75 °C. Real-time PCR assay protocol was performed using *SYBR*^®^*Green* Real-Time PCR Master Mixes (Roche, Germany) as described by our previous studies^32^. PCR primers were ACCACCATGGAGAAGGCTGG and CTCAGTGTAGCCCAGGATGC (GAPDH); CCTCTGGTCTTCTGGAGTACC and ACTCCTTCTGTGACTCCAGC (IL-6); GTGCCTATGTCTCAGCCTCT and TGGTTTGTGAGTGTGAGGGT (TNF-α); ACCTTCCAGGATGAGGACATGA and CTAATGGGAACGTCACACACCA (IL-1β). The specific genes were normalized to the level of GAPDH in each individual sample.

### Neurosphere radiant migration

Primarily cultured neurospheres were obtained from twenty C57BL/6 embryonic day 14 (E14) mice and kept as stable neurosphere cell lines. After the second passage (P2), neurospheres from cell lines were selected to perform migration assay. Six groups of neurospheres were treated with different proteins, including a control group (basic neural stem cell culture medium), a proBDNF (100 ng/mL) group, a mBDNF (100 ng/mL) group, a sheep anti-proBDNF antibody (10 μm/mL) group, a mouse anti-proBDNF antibody (10 μg/mL) group and a mouse anti-proBDNF antibody (10 μm/mL) supplemented with proBDNF (100 ng/mL). According to previous studies with some adjustments^33^, we picked the single neurosphere with the similar diameter of 500–600 μm and plated the neurospheres in the 24-well plates coated with Matrigel. Neurospheres were easily attached to the coating and cultured in different culture mediums for 48 hours, 37 °C, 5% CO_2_. All of the cell culture mediums were serum free that optimally inhibited neurospheres differentiation during our observed period of time (48 h). The single neurosphere migrated radiantly and formed a specific neural stem cell colony. Migrated distances were measured with Image-Pro Plus software.

### Statistical analysis

Data were shown by mean ± s.e.m. SPSS13.0 and GraphPad Prism 6 softwares wereused for the analysis of statistics and figures.Unpaired two-tailed Student’s t test, one-way ANOVA followed by Dunnett’s/Tukey’s Multiple Comparison post test or two-way ANOVA with Bonferroni’s Multiple Comparison post hoc test were used for mean comparisons, respectively. Differences were considered statistically significant at p < 0.05. N in the figure legend represents the number of animals used.

## Additional Information

**How to cite this article**: Luo, C. *et al*. Peripheral Brain Derived Neurotrophic Factor Precursor Regulates Pain as an Inflammatory Mediator. *Sci. Rep.* **6**, 27171; doi: 10.1038/srep27171 (2016).

## Supplementary Material

Supplementary Information
