# Supplementary material for: Peripheral Brain Derived Neurotrophic Factor Precursor Regulates Pain as an Inflammatory Mediator
Source: Sci Rep. 2016 Jun 2;6:27171. doi: 10.1038/srep27171 (PMC4890020; doi:10.1038/srep27171)
Supplement: Supplementary Information [file srep27171-s1.pdf]

Peripheral Brain Derived Neurotrophic Factor PrecursorRegulates Pain as an Inflammatory Mediator

Running title: peripheral proBDNF regulates pain

Cong Luo[1#], Xiao-Lin Zhong[2#], Fiona H Zhou[3#], Jia-yi Li[3], Pei Zhou[1], Jun-Mei Xu[1],Bo Song[4],Chang-Qi Li[2],Xin-Fu Zhou[3], Ru-Ping Dai*[1]

Supplementary Information

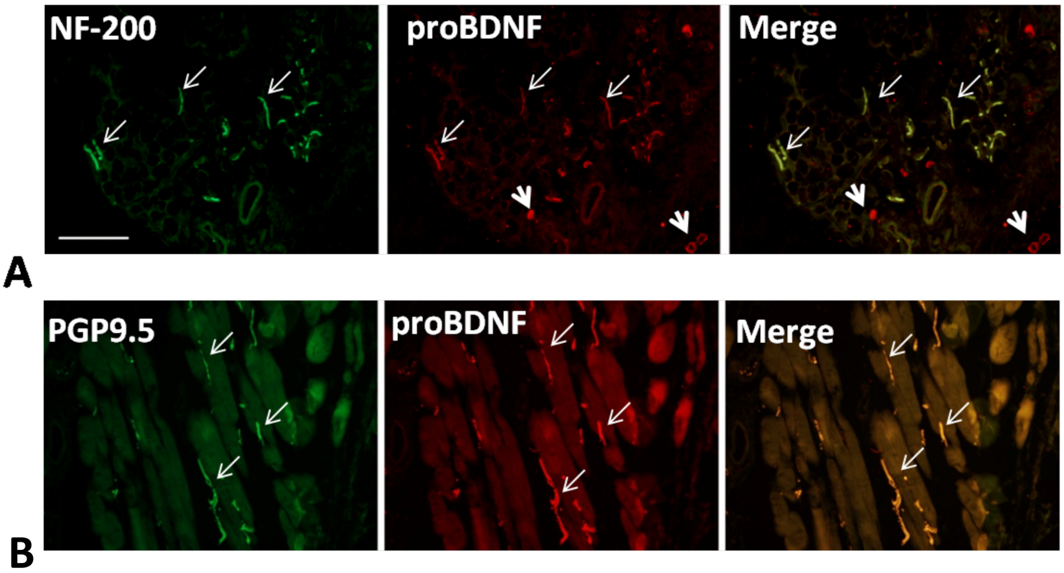

Supplementary Figure S1. Double labeling of proBDNF with nerve fiber markers in the footpad skin after formalin intra-plantar injection. Male Kunming-mice were conducted with unilateral intra-plantar injection with 10ul 5% formalin. At 3 h post-injection, mice were euthanized by over-dose chloral hydrate and processed for double-labeling immunofluorescence. (**A**) Co-localization of Neurofilament-200 (Green) with proBDNF (Red) after formalin hindpaw injection. Note the abundant co-localization of proBDNF with NF-200 (thin arrows). Some of

proBDNF positive staining was not expressed in the NF-200 positive staining (thick arrows). (**B**) Double labeling of PGP9.5 (Green) and proBDNF (Red) showing that proBDNF is co-localized with PGP9.5. Scale bar, 50μm, 3 replicates, n=3 per group.

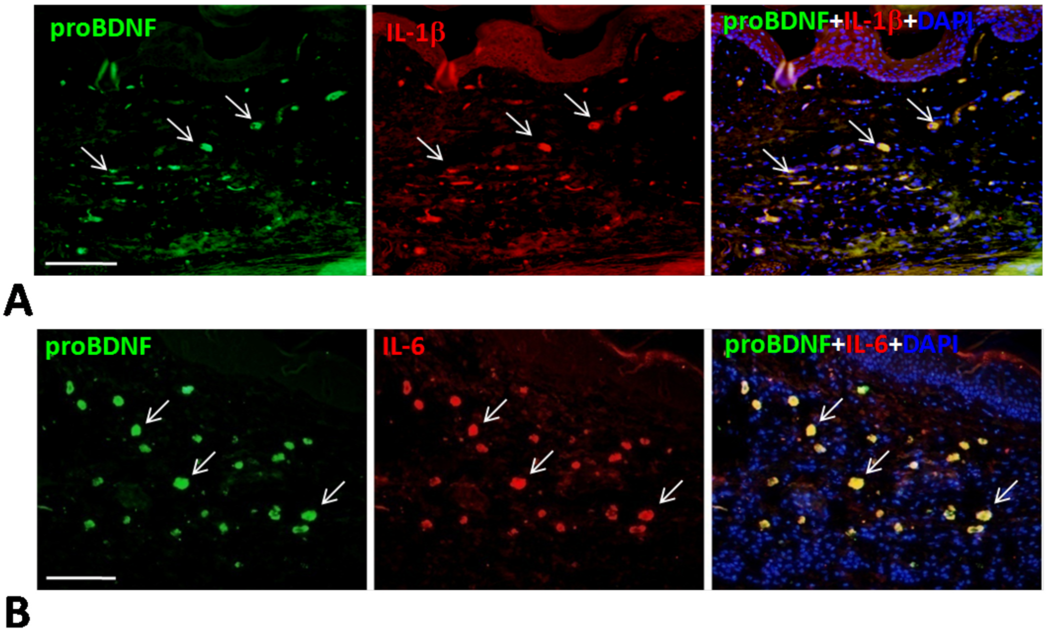

Supplementary Figure S2. **Co-localization of proBDNF with IL-1 β (A) and IL-6 (B) positive staining cells in response to formalin hindpaw injection**. Male Kunming-mice were subjected to 5% formalin intra-plantar injection and euthanized for immunofluorescence at 3h post-injection. Note that co-localization of IL-1β (**A**) and IL-6 (**B**) positive staining with proBDNF positive staining (Arrows). Scale bar, 50μm, 3 replicates, n=3 per group.

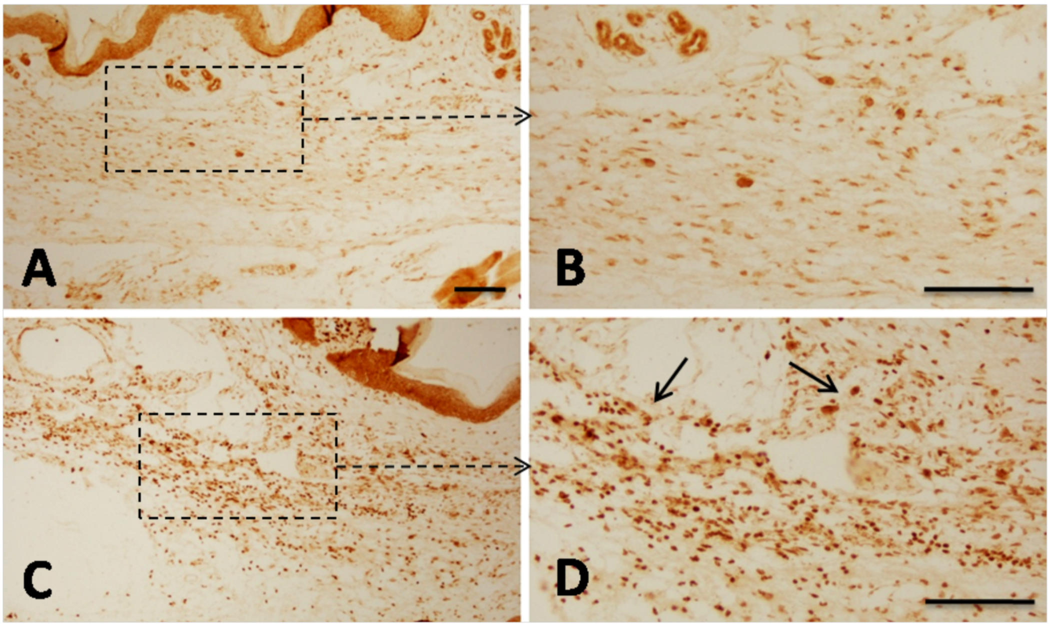

Supplementary Figure S3. **Representative immunohistochemistry of p75NTR in the local tissue in the normal control or at 3 h after formalin intra-plantar injection**. In the normal control foot skin, p75NTR immunoreactivity appeared to be localized in the nerve-fiber like structures in the subcutaneous layers (**A** and **B**). After formalin intra-plantar injection, p75NTR immunoreactivity was dramatically increased and mainly expressed in the inflammatory cells (Arrows, **C** and **D**). Scale bar, 50μm, 3 replicates, n=3 per group.

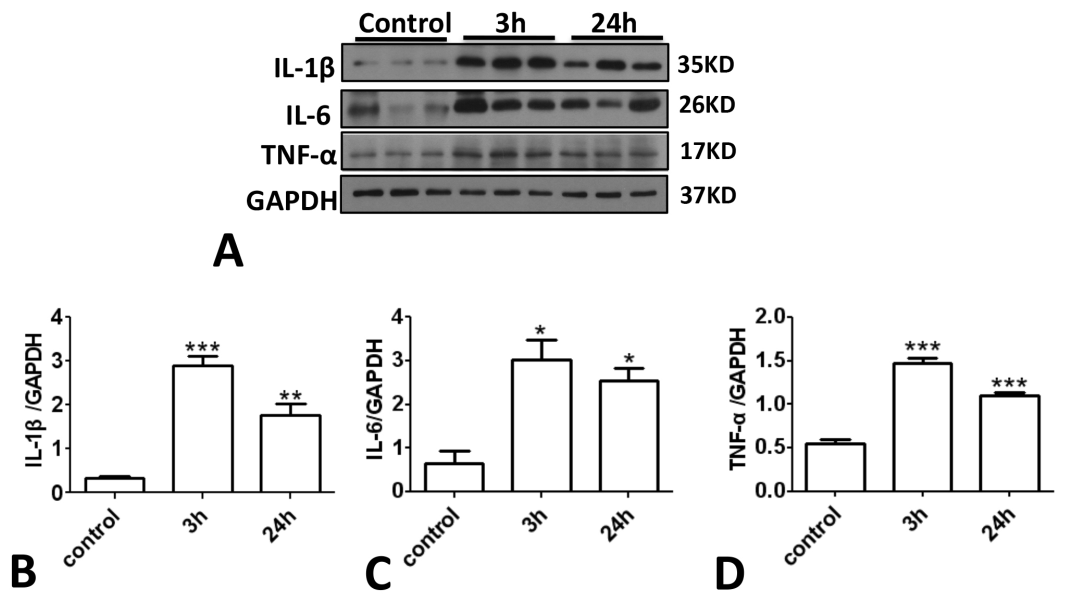

Supplementary Figure S4. **Western blot of IL-1β, IL-6 and TNF-a at 3 h and 24 h after proBDNF protein (0.25ug) intra-plantar injection into mice.** (**A**) Representative immunoblot of IL-1β, IL-6 and TNF-a after proBDNF protein injection. (**B-D**) Statistical analysis of IL-1β (**B**), IL-6 (**C**) and TNF-a (**D**) expression (*p<0.05, **p<0.01, ***p<0.001 versus control, one-way ANOVA followed by Dunnett's Multiple Comparison post hoc test, n=3 per group).
